# Renal Tubular HIF-2α Expression Requires VHL Inactivation and Causes Fibrosis and Cysts

**DOI:** 10.1371/journal.pone.0031034

**Published:** 2012-01-27

**Authors:** Ruth E. Schietke, Thomas Hackenbeck, Maxine Tran, Regina Günther, Bernd Klanke, Christina L. Warnecke, Karl X. Knaup, Deepa Shukla, Christian Rosenberger, Robert Koesters, Sebastian Bachmann, Peter Betz, Gunnar Schley, Johannes Schödel, Carsten Willam, Thomas Winkler, Kerstin Amann, Kai-Uwe Eckardt, Patrick Maxwell, Michael S. Wiesener

**Affiliations:** 1 Interdisciplinary Centre for Clinical Research, University Erlangen-Nuremberg, Erlangen, Germany; 2 Department of Nephrology & Hypertension, University Erlangen-Nuremberg, Erlangen, Germany; 3 Rayne Institute, University College London, London, United Kingdom; 4 Department of Nephrology, Charité, University Medicine Berlin, Berlin, Germany; 5 Hopital Tenon, UMR_S 702, INSERM/University of Paris 6, Paris, France; 6 Institute of Vegetative Anatomy, Charité, University Medicine Berlin, Berlin, Germany; 7 Department of Forensic Medicine, University Erlangen-Nuremberg, Erlangen, Germany; 8 Department of Genetics, University Erlangen-Nuremberg, Erlangen, Germany; 9 Department of Pathology, University Erlangen-Nuremberg, Erlangen, Germany; Cincinnati Children's Hospital Medical Center, United States of America

## Abstract

The Hypoxia-inducible transcription Factor (HIF) represents an important adaptive mechanism under hypoxia, whereas sustained activation may also have deleterious effects. HIF activity is determined by the oxygen regulated α-subunits HIF-1α or HIF-2α. Both are regulated by oxygen dependent degradation, which is controlled by the tumor suppressor “von Hippel-Lindau” (VHL), the gatekeeper of renal tubular growth control. HIF appears to play a particular role for the kidney, where renal EPO production, organ preservation from ischemia-reperfusion injury and renal tumorigenesis are prominent examples. Whereas HIF-1α is inducible in physiological renal mouse, rat and human tubular epithelia, HIF-2α is never detected in these cells, in any species. In contrast, distinct early lesions of biallelic VHL inactivation in kidneys of the hereditary VHL syndrome show strong HIF-2α expression. Furthermore, knockout of VHL in the mouse tubular apparatus enables HIF-2α expression. Continuous transgenic expression of HIF-2α by the Ksp-Cadherin promotor leads to renal fibrosis and insufficiency, next to multiple renal cysts. In conclusion, VHL appears to specifically repress HIF-2α in renal epithelia. Unphysiological expression of HIF-2α in tubular epithelia has deleterious effects. Our data are compatible with dedifferentiation of renal epithelial cells by sustained HIF-2α expression. However, HIF-2α overexpression alone is insufficient to induce tumors. Thus, our data bear implications for renal tumorigenesis, epithelial differentiation and renal repair mechanisms.

## Introduction

Mammalian cells require oxygen for energy homeostasis and thus for maintenance of cellular function and integrity. On the molecular level, adaption to reduced oxygen concentrations (hypoxia) depends on the activation of the Hypoxia-inducible Factor (HIF), which enables critical processes such as glycolysis, angiogenesis and erythropoiesis [1]. HIF is a transcriptional heterodimer, consisting of a constitutive ß-subunit and an oxygen sensitive α-subunit, HIF-1α or HIF-2α. Both α-subunits are regulated similarly [2], mainly by oxygen dependent hydroxylation leading to ubiquitination and proteasomal destruction [3]. However, knockout experiments [4], [5], tissue expression patterns [6], [7] and target gene specificity [8], [9] indicate isoform specific roles at least to some extent. Of note, in hypoxic rat kidneys HIF-1α and HIF-2α display a strikingly separate expression pattern. The former shows expression in tubular epithelia, whereas the latter shows expression in interstitial and glomerular cells [6].

For a number of reasons, the kidney has played a seminal role in understanding oxygen sensitive gene regulation. Despite a high oxygen transport rate to the kidney, oxygen tensions are very heterogeneous and in part lower as 10 mmHg [10]. Teleologically this may explain why the prototype of oxygen regulated genes, erythropoietin (EPO), is mainly induced in the kidney. Acute renal failure by ischemia-reperfusion injury is greatly attenuated if pharmacological preconditioning with HIF stabilizers is performed [11], [12]. Finally, the recognition component of the oxygen responsive ubiquitin ligase complex, the von Hippel Lindau (VHL) tumor suppressor [13], is a gatekeeper for growth control of tubular epithelial cells in the kidney [14]. In humans, biallelic inactivation of VHL leads to the development of renal cell carcinoma (RCC) of the clear cell type, which occurs in the hereditary VHL syndrome as well as in sporadic RCC. In mouse tissue specific VHL knockout was not found to induce tumors, but generated cysts either alone [15] or in conjunction with a PTEN knockout [16].

Human clear cell RCCs typically show global oxygen independent activation of HIF-1α and HIF-2α [17], [18]. Stabilization of HIFα subunits in early lesions of human RCC may be a decisive step in renal tumorigenesis [19]. Experimental studies have shown that HIF-2α, and not HIF-1α, appears to be the decisive subunit mediating tumorigenic features [20]–[23]. Mechanistically this may be due to a cellular proliferative effect of HIF-2α, whereas HIF-1α may have opposite effects [22], [24]. Nevertheless, overexpression of HIF-1a in murine proximal tubuli has recently been shown to lead to RCC [25]. Constant activation of HIF by genetic inactivation of VHL in tubular epithelia has further shown to induce renal fibrosis [26], which may indicate a common pathway of epithelial dedifferentiation.

In summary, HIF effects play an important role in the kidney, which can be beneficial or deleterious, depending on the setting and the timing. The specific roles of the different HIFα isoforms in this context are not well defined. Furthermore, there is no knowledge of the differential expression patterns of HIF-1α and HIF-2α in the human kidney. Experimental data originates mostly from VHL knockout studies in the mouse, where HIFα stabilization is an inevitable consequence. However, other HIF independent effects with oncogenic potential are known to be released when VHL is inactivated [14]. We therefore aimed to clearly define the physiological expression patterns of HIFα subunits in normal kidney epithelial cells, as well as in response to experimental VHL knockout and human VHL disease. Furthermore, we aimed to study the tumorigenic potential of tubular HIF-2α overexpression in a novel mouse model.

## Results

### Distinct expression patterns of HIF-1α and HIF-2α in hypoxic mouse and human kidneys

**Figure 1:**
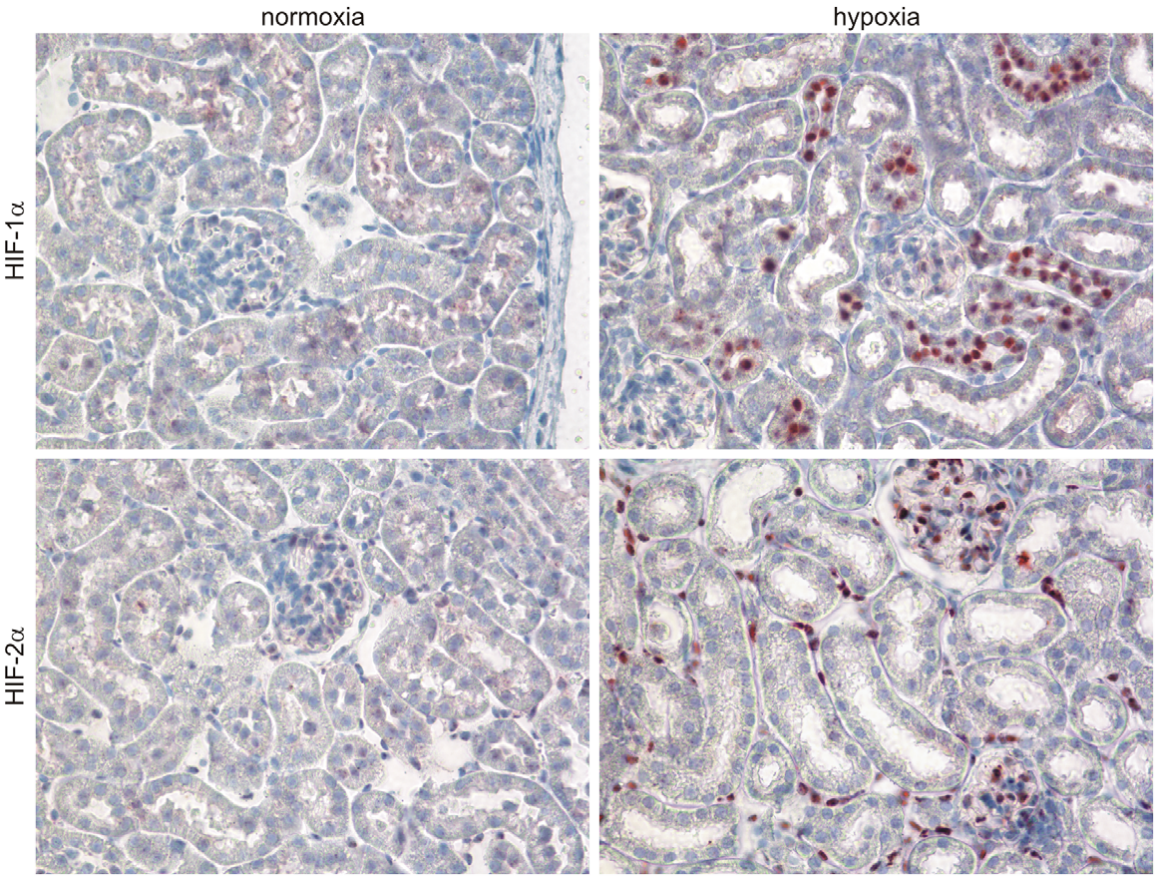
HIF-1α and HIF-2α expression in mouse kidney. The physiological expression pattern of HIF-1α and HIF-2α was analyzed by immunohistochemistry on kidney sections of normoxic and hypoxic (6 h, 7% O_2_) mice. HIF-1α expression was detected in the nuclei of renal tubular epithelial cells after hypoxic stimulation, whereas HIF-2α accumulated in interstitial and glomerular cells.

**Figure 2:**
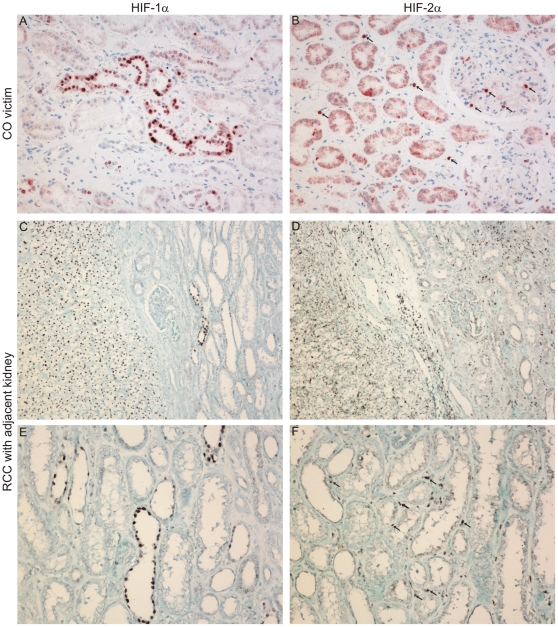
Expression of HIF-1α and HIF-2α in the human kidney. HIF-1α and HIF-2α expression was analyzed by immunohistochemistry in human kidneys. After carbon monoxide (CO) intoxication HIF-1α accumulation was detected in tubular epithelial cells (A) and HIF-2α was detected in interstitial cells and in the glomeruli, indicated by arrows (B). In kidneys of RCC patients HIF-1α and HIF-2α were detected in the tumor tissue as well as in the adjacent kidney (C and D). In the latter HIF-1α was found in tubular epithelial cells (E) and HIF-2α restricted to interstitial cells (F).

Identically to the predescribed situation in the rat, we see distinct expression patterns for the two HIFα subunits in mouse (Figure 1) and man (Figure 2). Hypoxic human kidneys were investigated from victims of carbon monoxide intoxication, where the tissues are severely hypoxic due to the reduced oxygen transport capacity of the haemoglobin and in kidney tissue adjacent to RCCs, where hypoxic microenvironment is often caused by expansive tumor growth. In all cases investigated, hypoxia leads to HIF-1α accumulation exclusively in tubular epithelial cells, whereas HIF-2α is stabilized in interstitial cells and in glomeruli. Hypoxia does not enable HIF-2α expression in renal tubular cells, in any species.

### Inducible VHL knockout in mouse renal tubular cells enables HIF-2α expression

**Figure 3:**
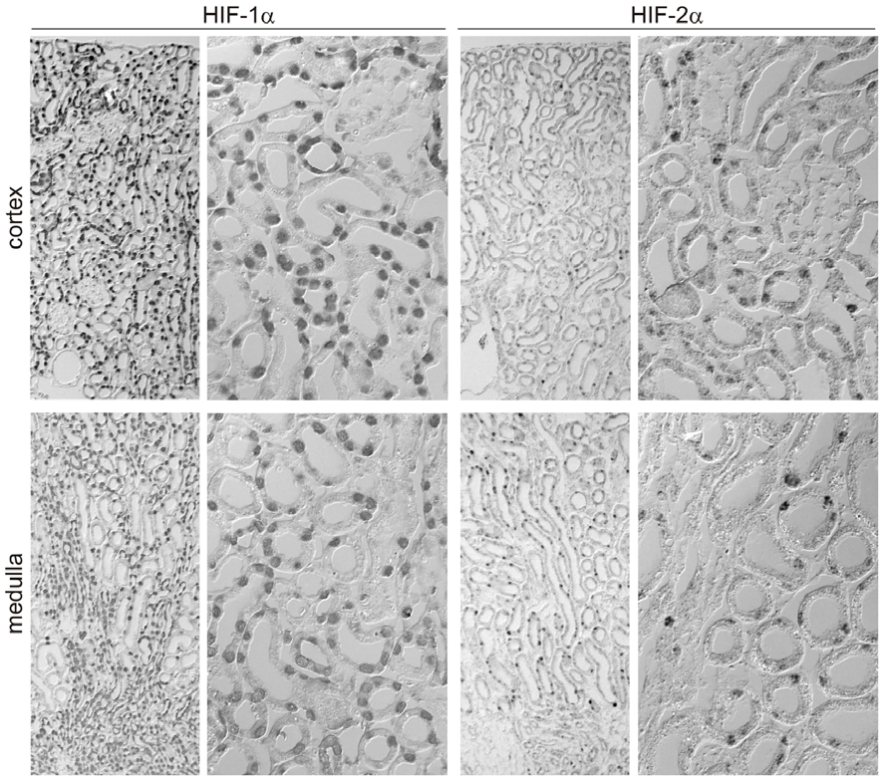
VHL knockout in mice releases tubular HIF-2α expression. HIF-1α and HIF-2α expression was analyzed by immunohistochemistry in the kidney cortex (upper panels) and medulla (lower panels) of inducible triple transgenic Pax8-VHL^−/−^ mice after 3 days of doxycyclin treatment (0.2 mg/ml in drinking water). HIF-1α as well as HIF-2α expression appears in renal tubular epithelial cells.

We next studied HIF expression in an inducible murine VHL knockout (VHL^−/−^) model, which was implied by the Pax8 promoter and confers an inducible Cre-driven knockout in the complete tubular system of the kidney [27], [28]. In these animals VHL expression is lost after doxycycline treatment. Control animals without doxycycline showed no expression of either HIFα subunit (data not shown). In contrast to the physiological HIF expression, the knockout mice showed tubular expression of HIF-2α (and HIF-1α) in the tubules upon a 3-day treatment with doxycyclin (Figure 3). Thus, it appears that VHL represses renal tubular HIF-2α specifically.

### Biallelic inactivation of VHL releases HIF-2α expression in distinct early lesions of the distal tubule in kidneys of the human VHL disease

**Figure 4:**
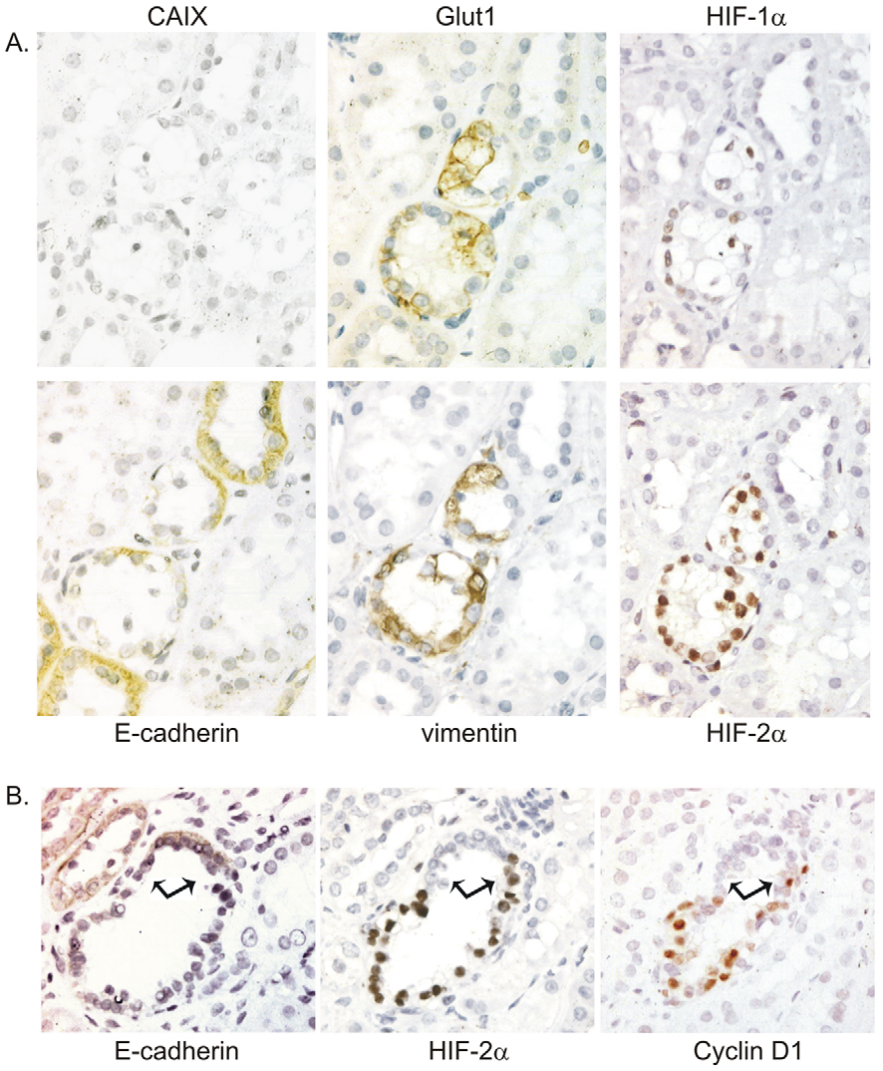
De-repression of HIF-2α in type II lesions of VHL disease kidneys. Early lesions of human VHL disease kidneys were stained for markers of epithelial dedifferentiation and HIF activation. A. Type II lesions are characterized by reduced E-cadherin expression, no expression of CAIX and little or no staining of HIF-1α. In contrast, strong staining for vimentin, Glut1 and HIF-2α can be seen. B. Type II lesions show pronounced upregulation of the HIF-2α target cyclin D1.

Previously, we reported multiple premalignant lesions in tubules of patients with VHL germline mutations, which were identified on the basis of carbonic anhydrase 9 (CAIX) expression [19]. We detected that these express HIF-1α, and had inactivation of the wild type VHL allele. Subsequently we demonstrated, that these foci in the distal tubule also displayed decreased expression of E-cadherin [29]. In the light of the mouse data, we re-examined the kidneys of VHL patients and observed additional foci of reduced E-cadherin labelling, which did not express CAIX and little or no HIF-1α, but did express HIF-2α (Figure 4 A). Thus there are (at least) two distinct classes of foci of VHL inactivation, which we now designate Type I (high CAIX, high HIF-1α) and Type II (no CAIX and high HIF-2α) foci (see table 1). We also stained for other markers, which are not expressed in normal renal tubules but do show expression in clear cell RCCs; the glucose transporter-1 (Glut1) and the intermediate filament vimentin. In line with the hypothesis that these lesions contain precancerous cells, the type II lesions stain clearly positive for vimentin and Glut1. Finally, in contrast to type I lesions the type II lesions show intense labelling for cyclin D1 (Figure 4 B), which has already been implicated to be a candidate of HIF-2 mediated tumorigenesis [22].

**Table 1:** Immunohistochemical labeling of type I and II foci in human VHL kidneys.

Protein	Type I foci	Type II foci
CAIX	+	−
E-cadherin	−	−
Glut1	+	+
HIF-1α	+	− (+)
HIF-2α	− (+)	+
Vimentin	+	+

Qualitative results for different stained proteins are shown as either not detectable (−) in early lesions or clearly detectable (+).

### Transgenic HIF-2α overexpression in renal tubular cells leads to renal fibrosis

**Figure 5:**
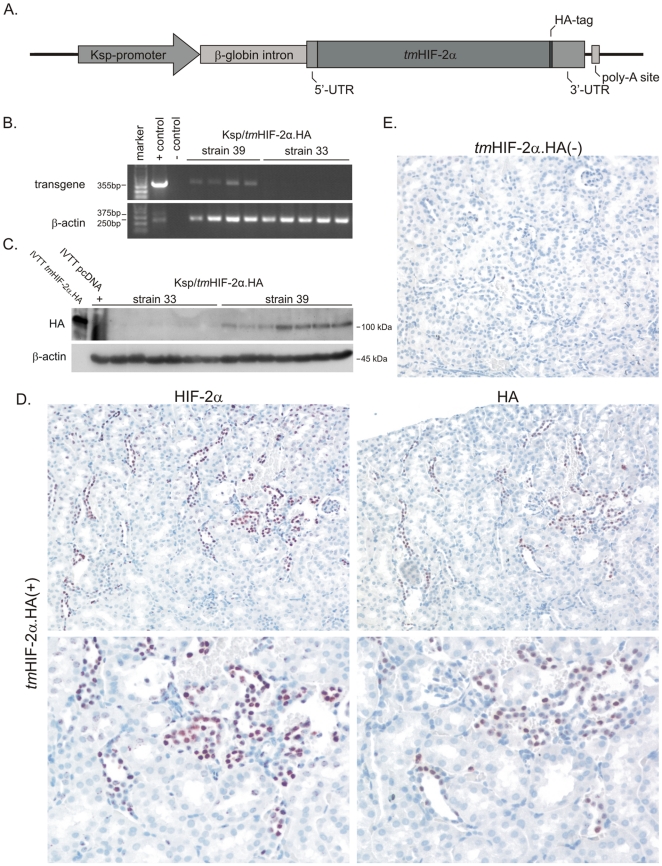
Generation of Ksp/*tm*HIF-2α.HA transgenic mice. A. Schematic representation of the pcKsp/*tm*HIF-2α.HA expression vector used for pronucleus injection consisting of a 1.3 kb Ksp-promoter fragment and an HA-tagged mutated HIF-2α triple mutant cDNA (Ksp, kidney specific; tm, triple mutant; UTR, untranslated region; HA-tag, influenza hemagglutinin epitope tag; poly-A site, poly-adenylation site). Injection of the Ksp/*tm*HIF-2α.HA construct successfully produced transgenic mice. Two of these were chosen and bred into homozygous strains, which was confirmed by genotyping for Ksp/*tm*HIF-2α integration and referred as Ksp/*tm*HIF-2α strain 33 and strain 39, respectively (data not shown). B. Analysis by RT-PCR detected transgenic *tm*HIF-2α.HA mRNA expression only in whole kidney RNA extracts from mice of strain 39. C. *tm*HIF-2α.HA protein expression was analyzed in whole kidney extracts from both strains by immunoblot. In parallel to the RNA expression, transgenic *tm*HIF-2α.HA protein was only expressed in strain 39. Based on these data strain 39 has been termed as *tm*HIF-2α.HA(+), whereas mice from strain 33 have been defined as *tm*HIF-2α.HA (−) and served as control strain, having the transgene integrated but not expressed. D. The localization of *tm*HIF-2α.HA in the mouse kidney was next analyzed by immunohistochemistry against HIF-2α and the HA-tag of the transgene on consecutive sections. Transgenic *tm*HIF-2α.HA expression was detected in tubular epithelial cells for HIF-2α (left hand panels) as well the HA-tag (right hand panels). E. Kidneys of the *tm*HIF-2α.HA(−) control strain were negative for both antibodies.

**Figure 6:**
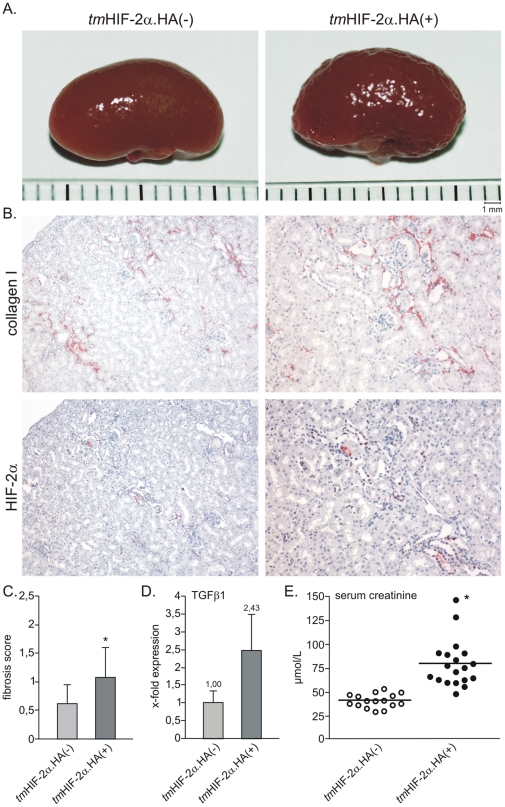
*tm*HIF-2α.HA(+) mice develop renal fibrosis and have impaired renal function. A. Representative photographs of the whole kidney from 15 month old *tm*HIF-2α.HA transgenic mice. The *tm*HIF-2α.HA(−) kidneys show a smooth surface, whereas the *tm*HIF-2α.HA(+) kidneys have an irregular surface structure. B. Immunohistological staining against collagen I shows increased interstitial deposition in close proximity to *tm*HIF-2α.HA expression in the transgenic kidney. C. Renal fibrosis was scored after SiriusRed staining of collagens. Increased fibrosis was detected in the kidneys of *tm*HIF-2α.HA(+) mice (results represent mean values of analyzed animals per strain with the error bars being standard deviation; *tm*HIF-2α.HA(−), n = 10; *tm*HIF-2α.HA(+), n = 11; * indicates p<0.05). D. The mRNA expression of the fibrosis associated gene TGFβ1 was analyzed by quantitative real-time PCR in whole kidney extracts. The expression was clearly up-regulated in the *tm*HIF-2α.HA(+) mice (results represent mean values with the error bars being standard deviation; n = 5 per strain). E. Renal function was analyzed by measurement of creatinine in the serum of the transgenic mice. *tm*HIF-2α.HA(+) mice have significantly increased plasma creatinine levels (* indicates p<0.05).

We generated a transgenic mouse model with constitutively stable and active HIF-2α derived from a cDNA (*tm*HIF-2α.HA) under the control of the kidney specific Ksp-promoter which enables mainly distal tubular expression (Fig. 5, S1 and S2). Kidneys of *tm*HIF-2α.HA(+) mice at the ages of 3, 6 and 9 months displayed no pathology regarding gross morphology and histology (data not shown). We therefore decided to let the mice grow to an age of 14–16 month. Figure 6 A shows representative photographs of the kidneys from *tm*HIF-2α.HA(−) and *tm*HIF-2α.HA(+) mice at this age. Obvious morphological differences can already be seen on the surface of kidneys from these two strains. The kidneys of *tm*HIF-2α.HA(−) control mice displayed a uniform smooth surface (Figure 6 A, left), whereas the kidneys of *tm*HIF-2α.HA(+) mice had an irregular and rough appearance (Figure 6 A, right). *tm*HIF-2α.HA(+) mice had a significantly reduced kidney weight as judged by kidney to body weight ratio (Figure S3 A). Histological analysis of the kidneys from aged animals was performed. The retracted organ surface implicated fibrotic events within the kidney. Therefore, total collagen as a fibrosis marker was stained by SiriusRed (Figure S3 B) and the level of tissue fibrosis was scored by a blinded analysis (Figure 6 C). The *tm*HIF-2α.HA(+) kidneys showed a strong increase of fibrotic tissue in comparison to *tm*HIF-2α.HA(−) kidneys. Furthermore, staining for collagen I revealed increased expression in *tm*HIF-2α.HA(+) mice (Figure 6 B, upper panel), whereas *tm*HIF-2α.HA(−) kidneys showed collagen I expression only to a minor extent (data not shown). Consecutive sections stained for collagen I and *tm*HIF-2α.HA revealed a clear spatial relationship between the transgenic HIF-2α expression and the areas of fibrosis (Figure 6 B). Real-time PCR for the fibrosis associated gene TGFb1 in total kidney RNA extracts showed strong upregulation in *tm*HIF-2α.HA(+) compared to *tm*HIF-2α.HA(−) kidneys (Figure 6 D). We additionally determined plasma parameters for renal function. Besides a slight increase in urea (data not shown), *tm*HIF-2α.HA(+) mice had significantly increased creatinine levels (Figure 6 E), confirming impaired renal function.

Real-time PCR analysis for HIF target genes from whole kidney lysates of the aged transgenic mice showed a strong induction of PHD3 and a moderate induction of TGFα and Glut1 in the *tm*HIF-2α.HA(+) mice (Figure S3 C). Since VEGF expression was induced as well and angiogenesis is believed to be a classical function of HIF, we histologically analyzed kidney capillarization in our transgenic mice. Kidney sections were stained for the endothelial marker MECA-32 by immunohistochemistry and the vascularization level was determined by counting positively stained capillaries in a blinded fashion. Compared to the *tm*HIF-2α.HA(−) control group, *tm*HIF-2α.HA expression led to a significant increase in kidney vascularization (Figure S3 D). However, EPO expression was not induced in *tm*HIF-2α.HA(+) mice and hematocrit not increased (data not shown).

### Transgenic HIF-2α overexpression in tubular cells leads to renal cyst formation

**Figure 7:**
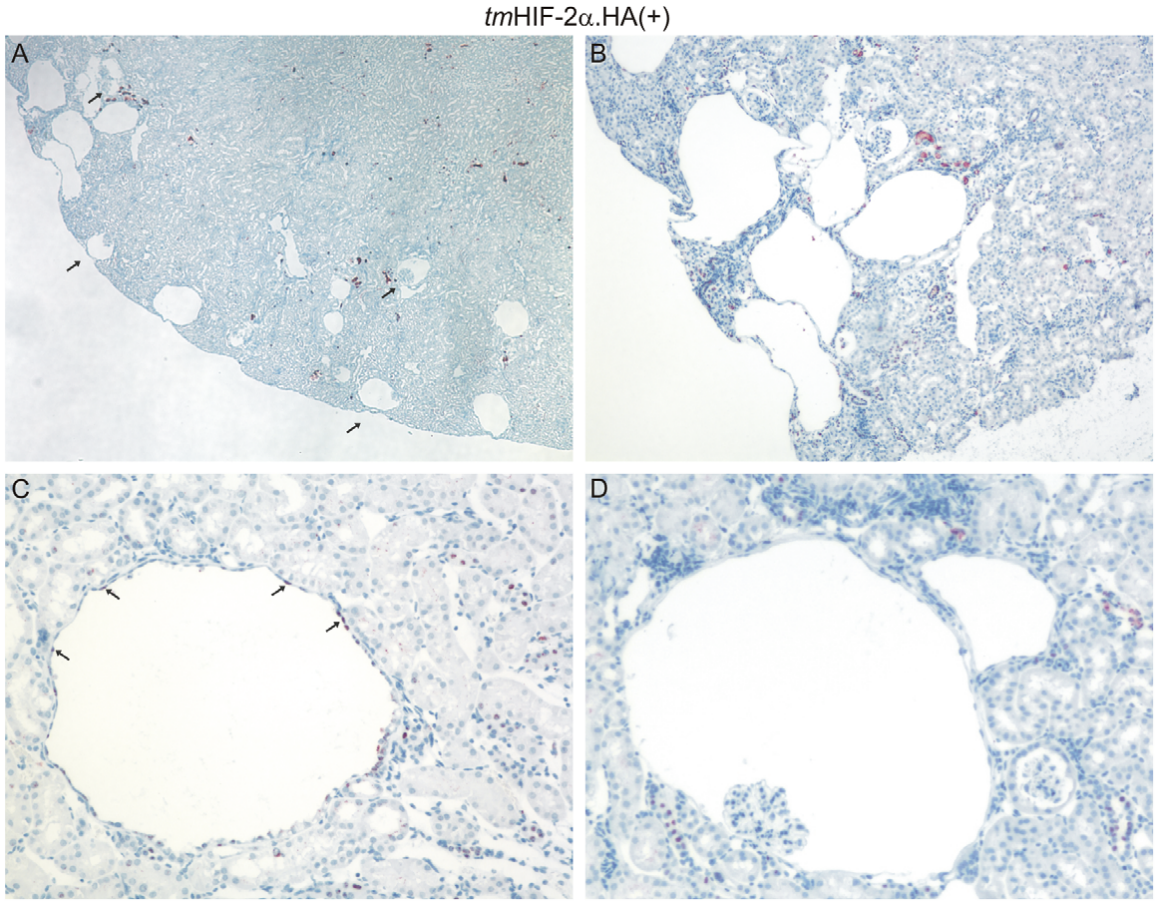
Renal cyst development in aged *tm*HIF-2α.HA(+) mice. 12 month and older *tm*HIF-2α.HA(+) mice develop multiple renal cysts, mainly in the kidney cortex. These partly form around glomeruli, where the glomerular tuft can be seen (arrows in A). Other cysts arise from distal tubular segments, where transgenic *tm*HIF-2α.HA expression can be detected in the epithelial lining of the cysts by immunohistochemistry against the HA-tag (arrows in C). In contrast, the glomerular cysts do not show transgene expression, with occasional positive labelling of tubular segments in the vicinity (D).

In addition to the fibrotic phenotype, the histological analysis of the *tm*HIF-2α.HA(+) transgenic mice revealed frequent formation of cysts in the renal cortex, which was not observed in the control strain (Figure 7). Higher magnification of the cysts showed the presence of a single epithelial cell layer lining the cystic lumen in all cases (Figure 7 C and D). Interestingly, there appeared to be two different types of cysts in the *tm*HIF-2α.HA(+) kidneys. Approximately half of the cysts derive from the proximal tubule immediately surrounding the glomeruli and often displaying the glomerulum inside the structure (glomerular cysts; Figure 7 A (marked by arrows) and D). The second group of cysts (termed tubular cysts) appear to derive directly from the distal tubules, since immunohistological staining against the HA-tag detected *tm*HIF-2α.HA expression in the nuclei of several cells of the cystic epithelium (Figure 7 C). In contrast, the epithelial cells lining the glomerular cysts show no nuclear labelling (Figure 7 D), whereas tubular segments in the vicinity stain positive for the transgene. Transgene expressing tubular segments appear to have normal numbers of cilia, where HIF-mediated reduction of cilia could have been a mechanism leading to cysts (Figure S4).

## Discussion

The transcription factor HIF is widely recognized as a critical mediator of many (patho)physiological processes and is currently under evaluation as a putative therapeutic target either by inhibition for tumor therapy or activation for organ protection. However, the spectrum of short and long term effects of HIF manipulation are difficult to foresee. Our study shows that constitutive aberrant overexpression of HIF-2α is sufficient to induce a complex type of kidney disease associated with tubular cyst formation, interstitial fibrosis and declining renal function, yet no development of RCC. These data bare implications for improved understanding of cellular control of HIF expression and its effects, as well as development and progression of kidney disease.

### Renal tubular HIF expression

We show that the distinct expression pattern for HIF-1a and HIF-2a in the kidney is stable across species and whatever stimulus is used (oxygen deprivation, application of prolyl hydroxylase inhibitors [11], [12] or neighbourhood to RCC). The molecular mechanism of differential control of HIFα expression remains elusive, where transcriptional, translational or posttranslational mechanisms could contribute. Importantly, when VHL is inactivated in renal tubular cells in mice or human VHL disease, we do see HIF-2α expression in these cells. The same has been described previously in another conditional VHL deletion model, although the authors did not compare the HIF-2α expression to microenvironmental stimulation [16]. This could mean, that VHL in some way specifically suppresses the expression of HIF-2α in tubular epithelial cells, which may have interesting implications for renal tumorigenesis. This is further supported by our findings in the early renal lesions of the human VHL disease. Type II foci show expression of HIF-2α, which is followed by upregulation of the proliferative target gene cyclin D1, which has been implicated in renal tumorigenesis before [22]. Accordingly, early studies have described a VHL dependent expression in RCC cells, which was more inclined to influence HIF-2α, rather than HIF-1α [18], [30].

### Renal cell carcinoma

The development of VHL associated clear cell RCC appears to be closely linked to HIF activation. It has been demonstrated that accumulation of HIF-1α and HIF-2α is a feature of very early tumorigenesis in kidneys derived from patients with the hereditary VHL syndrome, which can be verified already at a single cell level [19]. HIF-2α (and not HIF-1α) appears to be the decisive factor for growth initiation of experimental tumors. Functionally this may occur via pro-proliferative effects of HIF-2α, which may be mediated by cMyc signalling [24] and which can be verified in a subgroup of RCCs expressing HIF-2α only [31]. Thus, accumulating data has been published over the last decade implicating that HIF-2α activation is a seminal oncogenic hit in renal tumorigenesis as well as numerous other tumor entities, playing a further important role in tumor growth and behaviour [32]. We show that biallelic VHL inactivation releases HIF-2α expression in renal tubular cells. Therefore, this process could be an important event in the development of RCC. Nevertheless, our data also show that HIF-2α activation on its own is not sufficient to induce renal tumors in the mouse, which is similar to two further murine models who have deleted VHL in tubular cells [15], [16]. The lack of tumors in our and most other mouse models may be a result of the absence of crucial additional oncogenic events. Accordingly, it has been estimated that numerous mutational events are necessary to establish a malignant tumor, which is capable of enforcing itself in a hostile microenvironment [26]. However, HIF-1α overexpression in the proximal tubule seems sufficient to induce tumors in mice [25]. Thus, the particular segment of the renal tubule may also be of importance.

### Renal cystic disease

Regulation of renal tubular cell polarity and proliferation is strongly linked with the functional integrity of the primary cilium and ciliary defects often cause the development of renal cysts [33]. Intriguingly, recent data have shown that VHL and/or HIF play an important role in ciliary biology (for review see [34]). Consequently, two studies have demonstrated the development of renal cysts when VHL is inactivated in tubular cells with or without consecutive deletion of PTEN, respectively [15], [16]. It is not clear from these studies, which HIFα isoform mainly contributed to cyst development, although one study suspected HIF-2α to be the driving force, since simultaneous HIF-2α knockouts did not show cysts [15].

In human autosomal dominant polycystic kidney disease (ADPKD) HIF is also activated, but follows the “physiological” expression pattern of tubular HIF-1α and peritubular HIF-2α [35]. Of note, primary cystic diseases such as ADPKD rarely show the development of RCC, whereas acquired renal cysts and cysts in the hereditary VHL syndrome are regarded as precancerous lesions [34]. Whether the reason for these differences lie within HIF-2α expression of cystic epithelia remains speculative to date and is under investigation.

### Chronic kidney disease (CKD)

A large body of evidence exists showing that HIF activation can be beneficial in acute kidney injury models [11], [36], [37]. In CKD the situation is less clear although chronic hypoxia is considered to play a role in the development of progressive tubulointerstitial fibrosis and the development of CKD [38], [39]. Pharmacological HIF activation has been shown to be protective in chronic models of the remnant kidney by increase of peritubular angiogenesis [40], in the Thy1 nephritis [41] or models of diabetic nephropathy [42]. Nevertheless, HIF-1 appears to be an important mediator of CKD by chronic hypoxia, which is caused by EMT and subsequent fibrosis [26]. Furthermore, stable expression of HIF-1α in tubular epithelial cells seems to be sufficient to promote interstitial fibrosis [43]. Our mouse model has now shown that sustained overexpression of HIF-2α alone is sufficient to induce tubulointerstitial fibrosis and renal insufficiency, perhaps partly mediated by induction of TGFb1. The molecular mechanism of TGFb stimulation and subsequent fibrogenesis in our model is unclear to date. However, a number of fibrogenic pathways have been described which appear to be influenced after prolonged hypoxia in the kidney, such as PAI-1, CTGF and TGFß [39] or lysyl oxidases [44]. Interestingly, all these effects have so far been attributed to HIF-1α, rather than HIF-2α. However, since renal tubular cells do not physiologically express HIF-2α this effect may have been impossible to observe *in vivo*. The origin of renal fibrogenesis is discussed controversially in the literature (transdifferentiated epithelia [45] or extrarenal neurocrest derived fibroblasts [46]. Of note, in our model fibrogenesis clearly stems from a characteristic change in epithelial cells. The irregular and rough appearance of the transgenic kidneys probably stems largely from fibrotic retractions, as can be observed in severe human CKD. However, in some cases cysts could also cause the rough appearance wherever these structures bulge out of the surface. We believe that the former is the leading cause for this phenotype, since the latter only occurs regionally.

Our study was undertaken in order to improve the understanding of differential effects of HIF-1α and HIF-2α in the kidney. There are a number of limitations to our approach, such as the use of different promotors of our genetic models resulting in the targeting of differing nephron segments. Furthermore, it is unclear whether the observed phenotype is specific for.

HIF-2 activation, or whether it could also be achieved by overexpression of HIF-1, possibly by overlapping target genes. Finally, we have seen the described phenotype only in ageing mice, which suggests that the effects are rather mild in nature. Therefore, further studies will be needed to confirm and expand on our data. However, our data imply that the switch towards tubular HIF-2α expression is an important mediator of renal tumorigenesis, which can be achieved by VHL inactivation. The observed phenotype with renal cysts, tubulointerstitial fibrosis and CKD may share common pathways, but it could also be caused by parallel mechanisms. Of note, at least in mice HIF-2α appears not to be an independent oncogene. Hence, its overexpression may be an important early step in tumorigenesis but is not sufficient to induce RCCs, which may also account for the majority of early lesions in human VHL kidneys.

## Materials and Methods

### Generation of plasmid DNA, mouse strains and experimental animal protocols

For the generation of HIF-2α transgenic mice, a mutated and HA-tagged cDNA of HIF-2α was used as previously described (triple mutant *tm*HIF-2α; [8], [47]). The product is constitutively expressed and active, independent of oxygen levels. A 1.3 kb fragment of the kidney-specific (Ksp-) cadherin 16 promoter (described in [48]) was fused at its 3′-end to a β-globin intron and cloned into pcDNA3 generating the vector pcKsp/betaGl. Thereby the internal CMV promoter of the pcDNA3 backbone was deleted. The HA-tagged *tm*HIF-2α cDNA was cloned into pcKsp/betaGl at the 3′-site of the β-globin intron, generating the expression vector pcKsp/*tm*HIF-2α.HA. Pronucleus injection of the Ksp/*tm*HIF-2α.HA construct successfully produced transgenic mice in a C57Bl10xCBA/Ca hybrid background.

Conditional VHL knockout mice were generated by crossbreeding three different transgenic mouse lines: a) floxed VHL mice (Jackson Laboratories, Sacramento, CA) [49], b) LC-1 transgenic mice bearing Cre-recombinase expression under the control of the bidirectional P_tet_ promoter [50] and c) mice which express a reverse tetracycline-dependent transactivator under control of the Pax8 promoter [28] and are described previously [27]. For the induction of the VHL knockout, mice received 0.2 mg/ml doxycycline/5% glucose in the drinking water for 3 days.

All procedures involving mice were approved by the institutional review board (file number 31/25/04, University of Erlangen, Germany) and were in accordance with the National Institutes of Health guidelines. All data provided in the manuscript are pooled data from male and female mice.

### Renal function

Blood samples were taken immediately from sacrificed animals by aspiration from the heart. Plasma creatinine and urea were enzymatically measured using the Integra 800 (Roche).

### Collection of human tissues

RCC specimens were from historical collections [17], [19] collected from mostly radical tumor-nephrectomies and fixed in 4% paraformaldehyde (PFA). Written informed consent was obtained from each patient before nephrectomy. Paraformaldehyde fixed kidney specimens from victims of CO intoxication were from forensic medicine.

### Immunohistochemistry and antibodies

Paraffin sections (2 to 4 µm) were dewaxed in xylene and rehydrated in a series of ethanol washes. Immunohistochemistry was performed as described previously [51]. Detailed information for all primary antibodies is given in supplementary table S1. For detection of HIF-1α, HIF-2α and HA the signal amplification system (CSA) from DAKO (Glostrup, Denmark) was used according to the manufacturer's instructions and described in Rosenberger [6]. Biotinylated secondary anti-mouse, -rabbit, -sheep or -rat antibodies were from DAKO. Slides were partially counterstained with hematoxylin. Signals were analyzed with a Leica DMRB microscope (Leica, Bensheim, Germany). Photographs were digitally recorded by means of a Visitron system (Visitron, Puchheim, Germany).

### Protein extraction and immunoblot analysis

Cells were homogenized into extraction buffer (7 M urea, 10% glycerol, 10 mM Tris-HCl pH 6.8, 1% sodium dodecyl sulphate [SDS], 5 mmol/L dithiothreitol, 1 mM 4-(2-aminoethyl)-benzensulfonylfluorid [AEBSF], Complete Mini EDTA-free) using a T8 Ultra-Turrax homogenizer (IKA, Staufen, Germany) for 10 seconds at full speed. Extracts were quantified using the DC protein assay (BioRad, Munich, Germany). Proteins were resolved in 10% SDS polyacrylamide gels and transferred to Immobilon P (Millipore, Bedford, MA) overnight in blotting buffer (10 mM Tris, 100 mM glycine, 10% methanol, 0.05% SDS). Membranes were blocked with 3% fat-free dried milk in PBS with 0.1% Tween20 and probed with monoclonal antibodies against hemagglutinin tag (HA; 0.1 µg/ml; clone 3F10, Roche, Penzberg, Germany), and ß-Actin (0.48 ng/ml; clone AC-15, Sigma) and HRP-conjugated secondary goat-anti-rat (0.8 ng/ml; Dianova, Hamburg, Germany) and goat-anti-mouse antibodies (0.5 ng/ml; DAKO). Signals were visualized by chemiluminescence (Pierce, Rockford, IL). Displayed results are representative data of independent experiments.

### Cell culture and transient transfection assays

HeLa (human cervical cancer cell line), COS-7 (monkey kidney fibroblast cell line), HEK293 (human embryonic kidney fibroblast cell line) and OK (opossum renal tubular cell line) cells were from the European Cell Collection (ECACC). All cell lines were cultured in DMEN, 10% fetal calf serum, 100 U/ml penicillin, 0.1 mg/ml streptomycin (all from PAA).

For 24 h overexpression of triple-mutant HIF-2α, cells were seeded in 10 cm dishes and transfected with jetPei transfection reagent (Polyplus, New York, U.S.A.) following the manufacturer's instructions with 10 µg of the plasmid pcKsp/*tm*HIF-2α.HA or CMV promoter driven pc*tm*HIF-2α.HA ([52], [47]) as positive control and pcDNA3 empty vector as negative control.

For luciferase reporter assays cells were seeded in 24-well plates and transfected at a confluency of ∼50–60% with 0.5 µg of the 6xHRE luciferase reporter plasmids or 0.5 µg of the EPO-enhancer reporter plasmid and 0.1 µg pCMV-β-galactosidase expression vector (Stratagene, La Jolla, CA) using jetPei transfection reagent. For HIF-2α overexpression 50 ng of the expression vectors pcKsp/tmHIF-2α.HA or an equimolar amount of the empty vector pcKsp/betaGl was co-transfected. Luciferase activities were determined using the Luciferase Assay Reagent (Promega, Madison, WI) and normalized to β-galactosidase expression.

### *In vitro* generation of recombinant protein

Recombinant *tm*HIF-2α.HA protein was generated from the expression vector pc*tm*HIF-2α.HA by using the TNT® Quick coupled transcription/translation system (Promega) according to manufacturer's instruction. pcDNA3 empty vector was used as a negative control.

### RT-PCR and quantitative real-time PCR

Total RNA extracts were prepared by the use of RNAzol B (Biogenesis, Poole, U.K.) according to manufacturer's instructions. cDNA was generated using Superscript II RT (Invitrogen) according to manufacturer's instructions from 200 ng of total RNA.

The mRNA expression of Ksp-promotor driven *tm*HIF-2α.HA in the kidneys of transgenic mice was determined by RT-PCR using the following primers: mHIF2Eco-fw 5′-CGATGAATTCACCCAAAAATCTATGAG-3′; HA-tag-rev 5′-GTAGTCTGGGACGTCGTATGG-3′.

The mRNA expression of PHD3 and VEGF was determined by quantitative real-time PCR in duplicates with the Power SYBR Green PCR Mastermix (Applied Biosystems) according to manufacturer's instructions. Normalization was to HPRT housekeeping gene and fold-expression level was calculated using the ΔΔct method. The following primers were used: PHD3 fw 5′-CTATGTCAAGGAGCGGTCCAA-3′, PHD3 rev 5′-GTCCACATGGCGAACATAACC-3′; VEGF fw 5′-CAGGCTGCTGTAACGATGAA-3′, VEGF rev 5′-TATGTGCTGGCTTTGGTGAG-3′; HPRT fw 5′-GTTGGATACAGGCCAGACTTTGT-3′, HPRT rev 5′-CCACAGGACTAGAACACCTGC-3′.

The mRNA expression of Glut1, TGF-α and TGF-β1 was determined by quantitative real-time PCR in duplicates with the Taqman Gene Expression System (Applied Biosystems) according to manufacturer's instructions. Normalization was to β-2 microglobulin housekeeping gene and fold-expression level was calculated using the ΔΔct method. The following Taqman Gene Expression Assays were used: Glut1 Assay ID Mm01192270_m1; TGF-α Assay ID Mm00446231_m1; TGF-β1 Assay ID Mn00441727_g1; β2-m Assay ID Mm00437762_m1.

### Determination of fibrosis and vascularization score

The fibrosis level was quantified after SiriusRed staining of kidney sections. The score was determined by the percentage of staining in 15 randomly selected optical fields per kidney section at a 200× magnification according to the following ranking: 0% - score 0; 1–25% - score 1, 25–50% - score 2; 50–75% - score 3, more than 75% - score 4. Vascularization was quantified by counting signals in 15 randomly selected optical fields per kidney section at a 200× magnification after immunohistochemical staining against MECA-32. The origin of the samples was blinded to the pathologist doing the scoring.

## Supporting Information

Figure S1 ***In vitro*** **analysis of the pcKsp/** ***tm*** **HIF-2α.HA expression vector.** A. Transient expression of *tm*HIF-2α.HA under control of the Ksp-promoter or CMV-promoter (positive control) and expression of the empty vector pcDNA3 (negative control) was analyzed in HeLa, COS-7, HEK293 and OK cells. Expression of *tm*HIF-2α.HA under standard promoter control occurred in all cell lines, whereas Ksp-promoter driven *tm*HIF-2α.HA is only expressed in the kidney epithelial cell line. B. Transactivation functionality of the pcKsp/*tm*HIF-2α.HA construct was analyzed by two HIF dependent luciferase reporter in renal tubular OK cells. Forced expression of the pcKsp/*tm*HIF-2α.HA construct induced 6xHRE and EPO-E reporter gene expression up to 3.4-fold (mean values of three independent experiments with the error bars being standard deviation; * indicates p<0.05).
(TIF)

Figure S2 ***tm*** **HIF-2α-HA is expressed in the distal tubule and the collecting duct.** Transgenic *tm*HIF-2α.HA expression is localized in the distal part of the renal tubule and the collecting duct shown by immunohistochemistry. HA staining colocalized with staining against the tubular segment markers NCC (A), 11βHSD (B) and AQP-2 (C) in the distal tubulus and the collecting duct.
(TIF)

Figure S3 **Analysis of kidneys from** ***tm*** **HIF-2α.HA overexpressing mice.** The kidneys of *tm*HIF-2α.HA(+) transgenic mice show a significantly reduced weight compared to the *tm*HIF-2α.HA(−) control animals (results represent mean values of analyzed animals per strain with the error bars being standard deviation; *tm*HIF-2α.HA(−) n = 16; *tm*HIF-2α.HA(+) n = 19; * indicates p<0.05). B. Renal fibrosis was analyzed by SiriusRed staining of connective tissue, mainly collagens. Kidneys of *tm*HIF-2α.HA(+) mice displayed strong fibrotic staining. C. HIF target gene activation was analyzed by quantitative real-time PCR of whole kidney extracts from Ksp/*tm*HIF-2α.HA transgenic mice at different ages. PHD3 expression was clearly increased in the kidneys of *tm*HIF-2α.HA(+) mice. TGFα, Glut1 and VEGF expression were moderately induced in the *tm*HIF-2α.HA(+) animals (results represent mean values of tested animals per strain with the error bars being standard deviation; n = 5 per strain). D. Vascularization of the transgenic kidneys was analyzed by endothelial MECA-32 staining and determination of the staining signals per mm^2^. *tm*HIF-2α.HA(+) mice displayed a stronger vascularization of their kidneys (cap, capillary; results represent mean values of analyzed animals per strain with the error bars being standard deviation; *tm*HIF-2α.HA(−), n = 9; *tm*HIF-2α.HA(+), n = 11; * indicates p<0.05).
(TIF)

Figure S4 **Cilia in kidneys from** ***tm*** **HIF-2α.HA overexpressing mice.** Cilia from *tm*HIF-2α.HA(+) transgenic mice were visualized with an anti-Acetylated Tubulin antibody (clone 6-11B-1, Sigma; sec. antibody AlexaFlour 594) and co-stained with an anti-NCC (Oregon Health & Science University; sec. antibody AlexaFlour 488) or anti-11βHSD (Millipore, Billerica, MA; sec. antibody AlexaFlour 488) antibody using the mouse on mouse Kit (M.O.M.-Kit) from Vector Laboratories (Burlingame, CA) according to the manufactures instructions. The figure shows representative data from old and young mice co-stained for Ac-Tubulin and NCC (A) or Ac-Tubulin and 11βHSD (B), respectively. All tubuli identified by the segment specific markers showed clearly stained cilia strongly suggesting that the overexpression of HIF-2α does not influence cilia formation.
(TIF)

Table S1 **Primary antibodies.** Primary antibodies used for immunohistochemistry are listed with inclusion of source and dilution.
(DOC)
