# Supplementary figures and images for: Renal Tubular HIF-2α Expression Requires VHL Inactivation and Causes Fibrosis and Cysts
Source: PLoS One. 2012 Jan 27;7(1):e31034. doi: 10.1371/journal.pone.0031034 (PMC3267769; doi:10.1371/journal.pone.0031034)

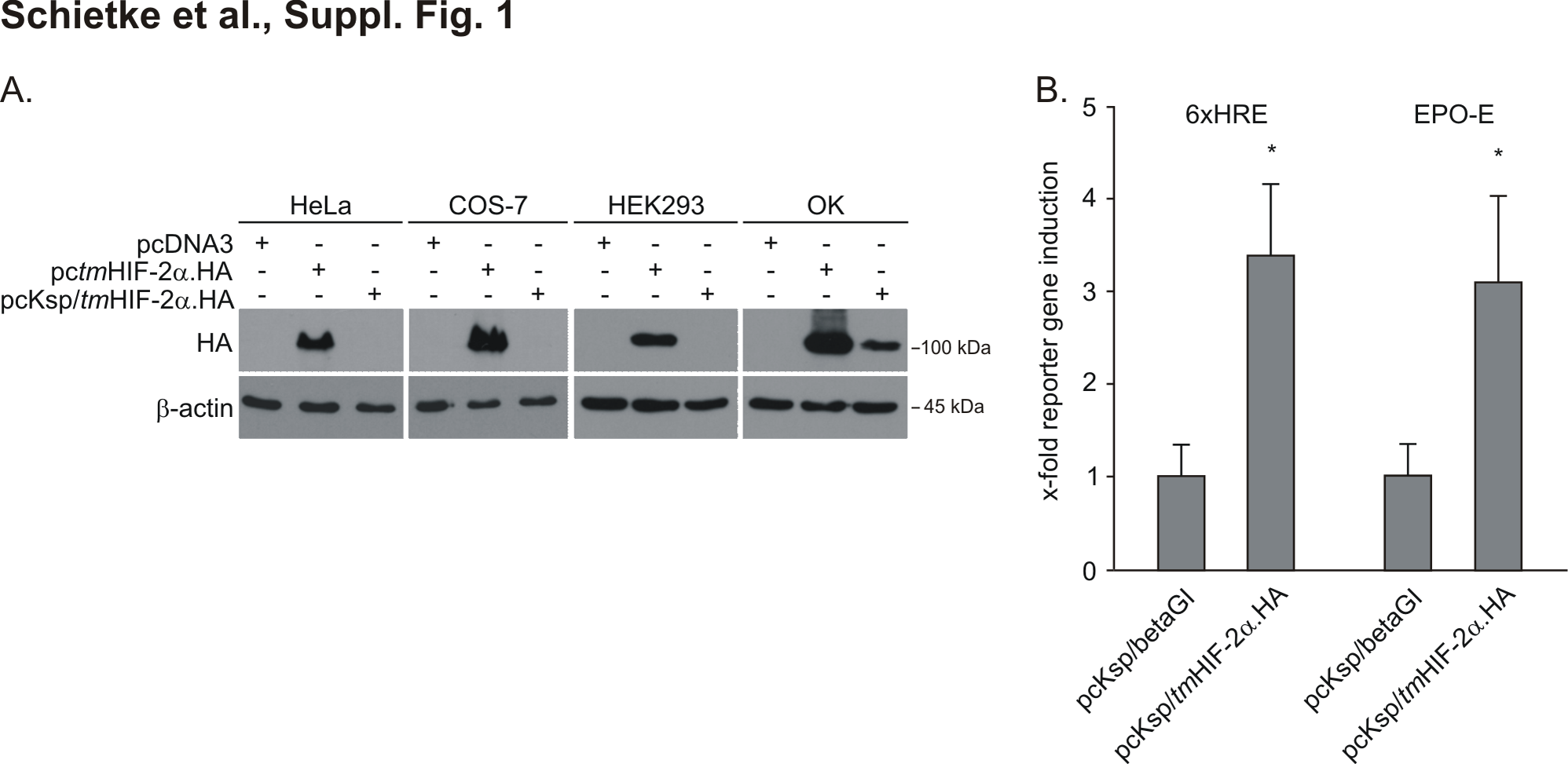

Supplement: Figure S1 — In vitro analysis of the pcKsp/ tm HIF-2α.HA expression vector. A. Transient expression of tmHIF-2α.HA under control of the Ksp-promoter or CMV-promoter (positive control) and expression of the empty vector pcDNA3 (negative control) was analyzed in HeLa, COS-7, HEK293 and OK cells. Expression of tmHIF-2α.HA under standard promoter control occurred in all cell lines, whereas Ksp-promoter driven tmHIF-2α.HA is only expressed in the kidney epithelial cell line. B. Transactivation functionality of the pcKsp/tmHIF-2α.HA construct was analyzed by two HIF dependent luciferase reporter in renal tubular OK cells. Forced expression of the pcKsp/tmHIF-2α.HA construct induced 6xHRE and EPO-E reporter gene expression up to 3.4-fold (mean values of three independent experiments with the error bars being standard deviation; * indicates p<0.05). (TIF) [file pone.0031034.s001.tif]

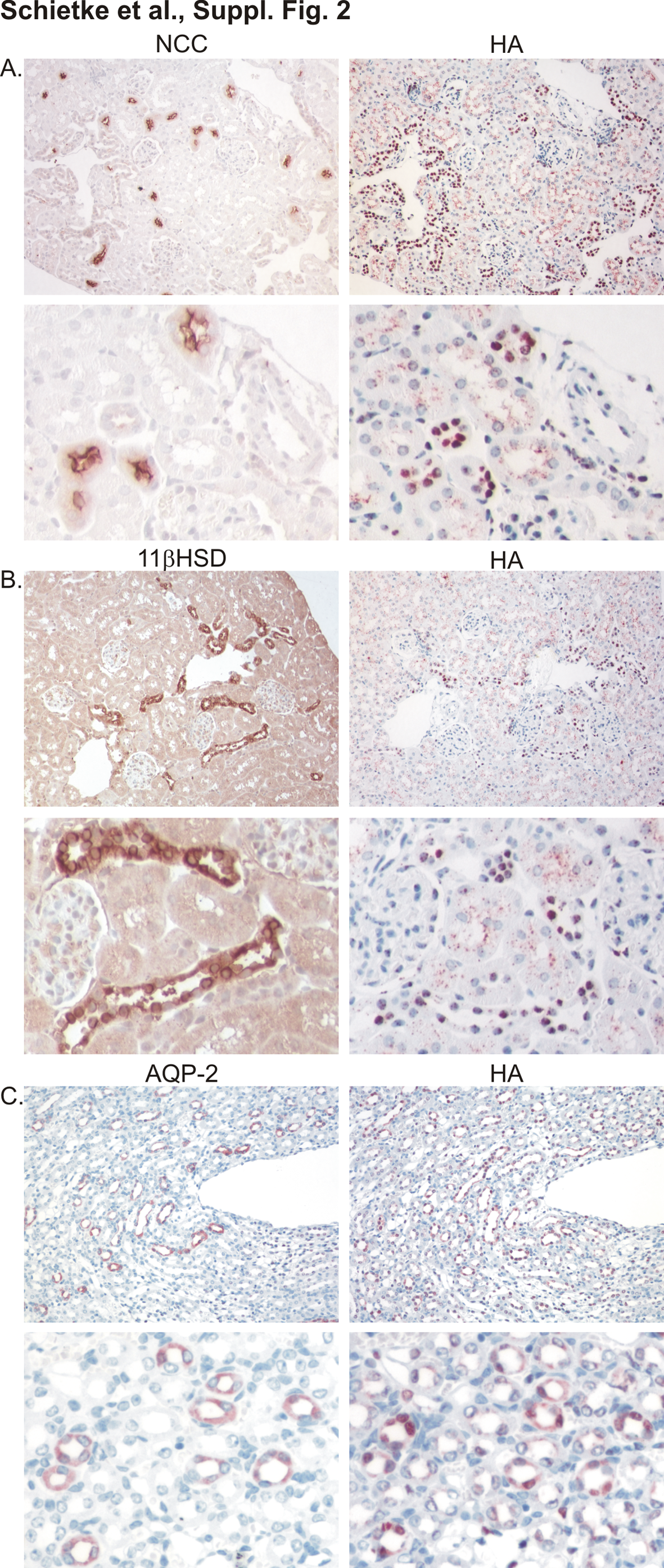

Supplement: Figure S2 — tm HIF-2α-HA is expressed in the distal tubule and the collecting duct. Transgenic tmHIF-2α.HA expression is localized in the distal part of the renal tubule and the collecting duct shown by immunohistochemistry. HA staining colocalized with staining against the tubular segment markers NCC (A), 11βHSD (B) and AQP-2 (C) in the distal tubulus and the collecting duct. (TIF) [file pone.0031034.s002.tif]

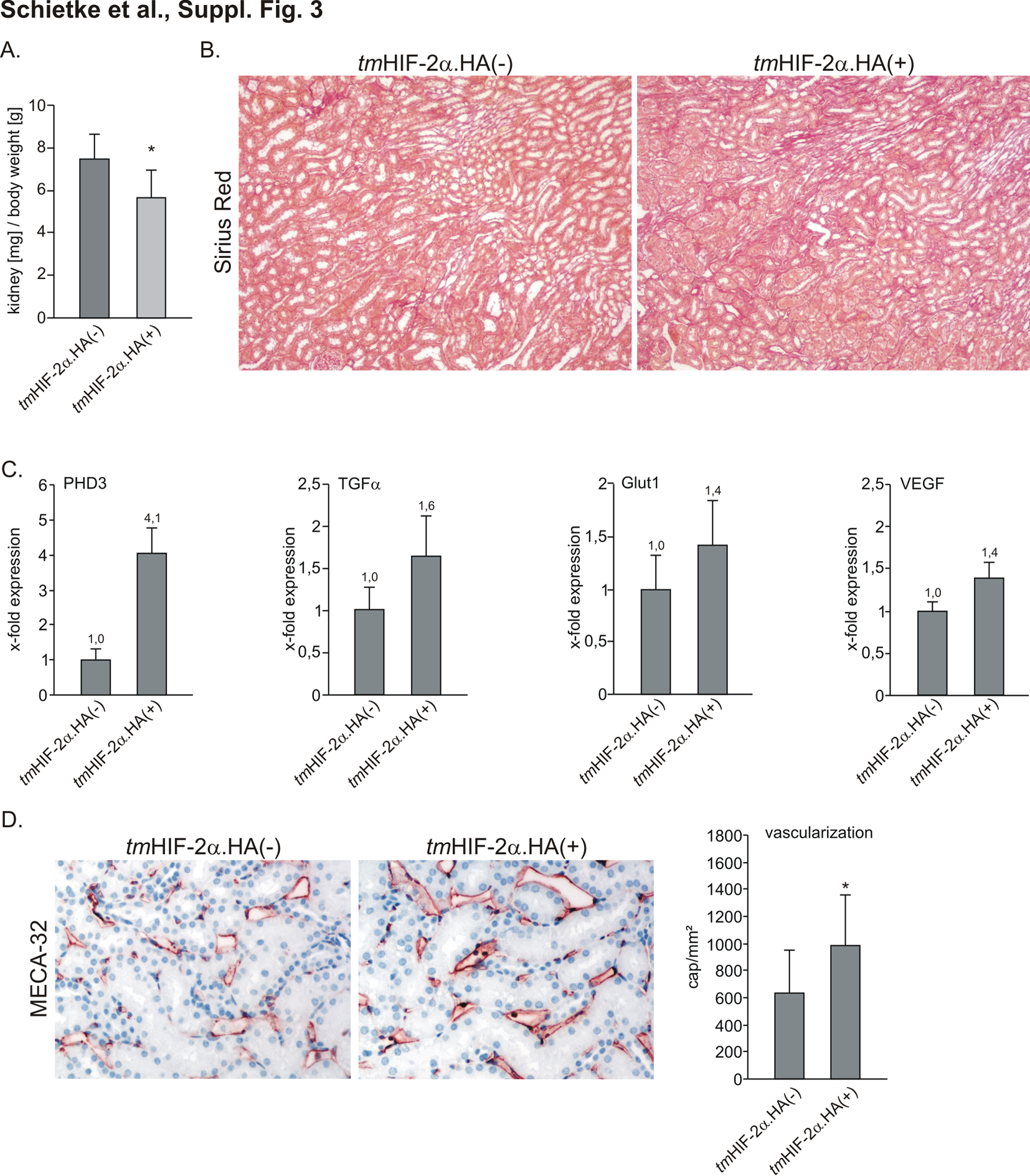

Supplement: Figure S3 — Analysis of kidneys from tm HIF-2α.HA overexpressing mice. The kidneys of tmHIF-2α.HA(+) transgenic mice show a significantly reduced weight compared to the tmHIF-2α.HA(−) control animals (results represent mean values of analyzed animals per strain with the error bars being standard deviation; tmHIF-2α.HA(−) n = 16; tmHIF-2α.HA(+) n = 19; * indicates p<0.05). B. Renal fibrosis was analyzed by SiriusRed staining of connective tissue, mainly collagens. Kidneys of tmHIF-2α.HA(+) mice displayed strong fibrotic staining. C. HIF target gene activation was analyzed by quantitative real-time PCR of whole kidney extracts from Ksp/tmHIF-2α.HA transgenic mice at different ages. PHD3 expression was clearly increased in the kidneys of tmHIF-2α.HA(+) mice. TGFα, Glut1 and VEGF expression were moderately induced in the tmHIF-2α.HA(+) animals (results represent mean values of tested animals per strain with the error bars being standard deviation; n = 5 per strain). D. Vascularization of the transgenic kidneys was analyzed by endothelial MECA-32 staining and determination of the staining signals per mm2. tmHIF-2α.HA(+) mice displayed a stronger vascularization of their kidneys (cap, capillary; results represent mean values of analyzed animals per strain with the error bars being standard deviation; tmHIF-2α.HA(−), n = 9; tmHIF-2α.HA(+), n = 11; * indicates p<0.05). (TIF) [file pone.0031034.s003.tif]

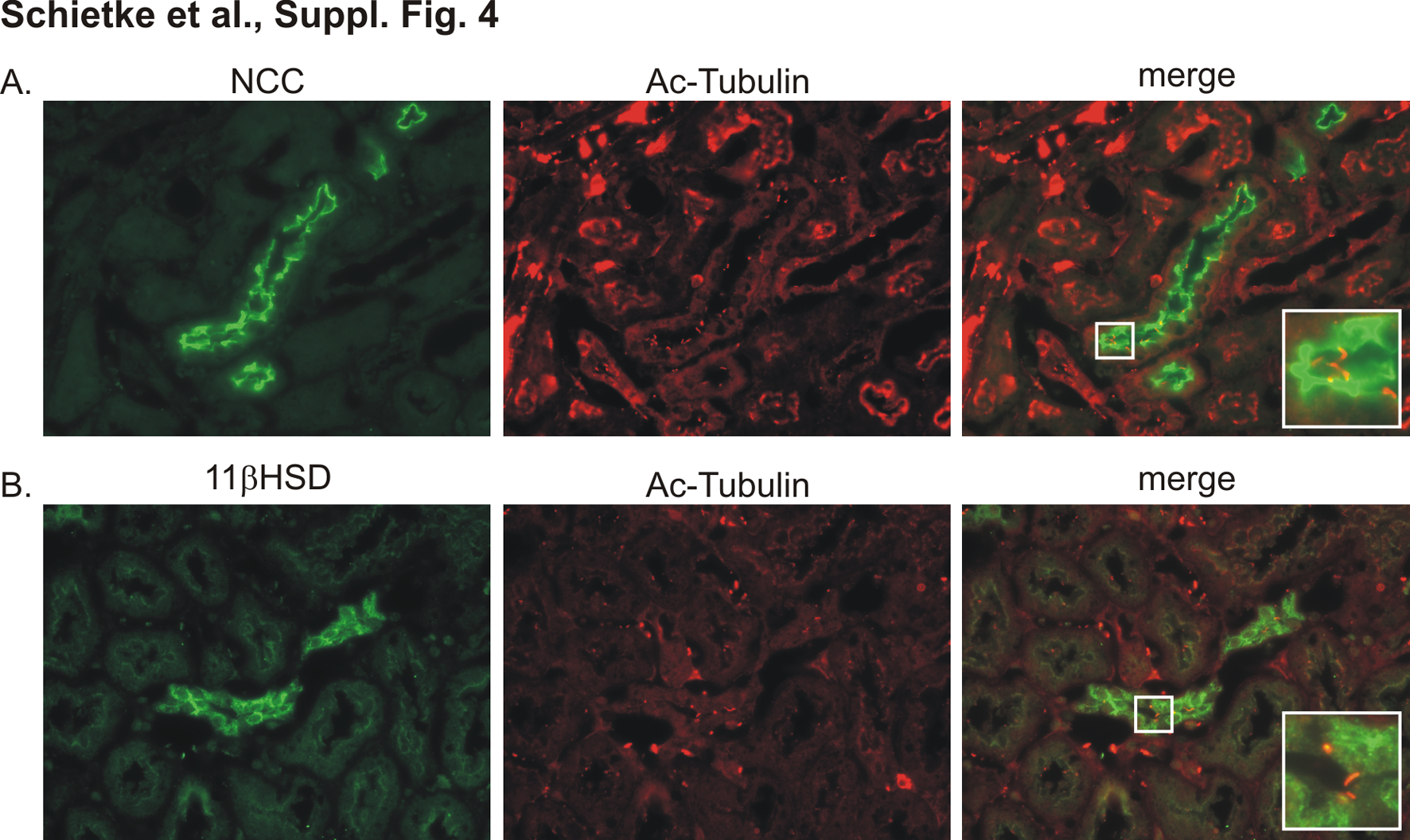

Supplement: Figure S4 — Cilia in kidneys from tm HIF-2α.HA overexpressing mice. Cilia from tmHIF-2α.HA(+) transgenic mice were visualized with an anti-Acetylated Tubulin antibody (clone 6-11B-1, Sigma; sec. antibody AlexaFlour 594) and co-stained with an anti-NCC (Oregon Health & Science University; sec. antibody AlexaFlour 488) or anti-11βHSD (Millipore, Billerica, MA; sec. antibody AlexaFlour 488) antibody using the mouse on mouse Kit (M.O.M.-Kit) from Vector Laboratories (Burlingame, CA) according to the manufactures instructions. The figure shows representative data from old and young mice co-stained for Ac-Tubulin and NCC (A) or Ac-Tubulin and 11βHSD (B), respectively. All tubuli identified by the segment specific markers showed clearly stained cilia strongly suggesting that the overexpression of HIF-2α does not influence cilia formation. (TIF) [file pone.0031034.s004.tif]
