# Supplementary material for: Renal Tubular HIF-2α Expression Requires VHL Inactivation and Causes Fibrosis and Cysts
Source: PLoS One. 2012 Jan 27;7(1):e31034. doi: 10.1371/journal.pone.0031034 (PMC3267769; doi:10.1371/journal.pone.0031034)
Supplement: Table S1 — Primary antibodies. Primary antibodies used for immunohistochemistry are listed with inclusion of source and dilution. (DOC) [file pone.0031034.s005.doc]

**Table S1: Primary antibodies.**

Primary antibodies used for immunohistochemistry are listed with inclusion of source and dilution.

| **Detected Protein** | **Primary antibody - Source** | **Origin** | **Dilution** |
| --- | --- | --- | --- |
| 11βHSD (11beta-hydroxysteroid dehydrogenase) | Millipore, Billerica, MA | sheep | 1:200 |
| AQP-2 (Aquaporin 2) | Novus Biologicals, Littleton, CO | rabbit | 1:500 |
| CAIX (Carbonic anhydrase 9) | clone M75, kind gift of S. Pastorekova, Bratislava, Slovak Republic | mouse | 1:50 |
| Collagen I | Abcam, Cambridge, U.K. | rabbit | 1:500 |
| Cyclin D1 | clone SP4, Abcam, Cambridge, U.K. | rabbit | 1:200 |
| E-cadherin | clone HECD-1, Abcam, Cambridge, U.K. | mouse | 1:100 |
| Glut1 (Glucose transporter 1) | DAKO, Glostrup, Denmark | mouse | 1:250 |
| HA (Hemagglutinin) | clone HA.11, Covance, Berkeley, CA | rabbit | 1:10.000 |
| HIF-1α | clone α67; Novus Biologicals, Littleton, CO | mouse | 1:10.000 |
| HIF-2α | PM9 obtained from rabbit immunized with a peptide containing amino acids 337 to 439 of mouse HIF-2α | rabbit | 1:50.000 |
| MECA-32 (Panendothelial cell antigen) | BioLegend San Diego, CA | rat | 1:50 |
| NCC (Na^+^-Cl^-^ cotransporter) | Oregon Health & Science University | rabbit | 1:500 |
| Vimentin | DAKO, Glostrup, Denmark | mouse | 1:1000 |
